# Navigation and closed-loop control of magnetic microrobot in plant vein mimic environment

**DOI:** 10.3389/fpls.2023.1133944

**Published:** 2023-03-08

**Authors:** Zhijie Huan, Jiamin Wang, Lan Zhu, Zhixiong Zhong, Weicheng Ma, Zhoufan Chen

**Affiliations:** ^1^ School of Electrical Engineering and Automation, Xiamen University of Technology, Xiamen, China; ^2^ Fujian Provincial Key Laboratory of Information Processing and Intelligent Control, Minjiang University, Fuzhou, China

**Keywords:** navigation, closed-loop control, microrobot, electromagnetic actuated system, micro-environment

## Abstract

In recent years, research on the manipulation and control of microrobot has gradually matured. In order to improve the intelligence of microrobots, navigation study also becomes an important research topic. In practice, microrobots could be disturbed by the flowing liquid when it moves in a microfluidic environment. As a result, the actual trajectory of microrobots will deviate from the intended one. In this paper, firstly, different algorithms for the navigation of microrobots in a simulated plant leaf vein environment are investigated. According to the simulation results, RRT*-Connect is then selected as the path planning algorithm with a relatively better performance. Based on the pre-planned trajectory, a fuzzy PID controller is further designed for precise trajectory tracking, which can effectively eliminate the random disturbance caused by micro-fluid flow during the motion and make it quickly recover to a stable movement state.

## 1 Introduction

Relative to the development of microrobotic-driven technology, a big number of researchers have investigated its application in biomedical therapies, including targeted drug delivery, vascular navigation, biomarkers, and cell delivery (Arcese et al., 2010; Ju et al., 2014; Kim and Ishiyama, 2014; Liu et al., 2017; Fan et al., 2018). Li et al. (2010) proposed a novel artificial hair receptor prototype for a hopping minirobot sensing system with a high aspect ratio of aligned polyvinylidine (PVDF) micro/nanofiber arrays. It also proves the feasibility of the thermal direct fiber stretching technology on PVDF materials and the application prospect of the produced fibers as a micro/micro-biological robot sensing system. The reliable response and the good sensitivity of micro/nano PVDF fibers to pressure changes and various flows were verified. It shows that artificial hair cell receptors are very promising for wind properties and environmental vibration detection, which are essential for jumping microrobot sensing systems. Chen et al. (2020) designed a magnetically driven thumb-shaped and disc-like bilayer (chitosan and calcium alginate hydrogel) drug-loaded mini-robot with high mobility and motion precision. It has advanced mobility, pH sensitivity, and sustained drug release capability. The results of this work pave the way for targeted therapy for intestinal cancer after oral drug administration.

The microfluidic environment in human or plants could be complex for the manipulation of microrobot. To achieve the autonomous navigation movement of microrobots, the manipulation process should be combined with rapid path planning algorithm. Meng et al. (2019) used width-first search and genetic algorithms to generate robotic navigation reference tracks and proposed a navigation controller combined with slide control, backward control, and perturbation compensation to realize the targeted transport of tumor drugs in mouse liver blood vessels. Liu et al. (2020) applied Informed-RRT* for path planning for the spiral micro-swimming robot in a chaotic environment. Kinematical and nonintegrity constraint of the spiral micro-swimming robot are proposed, which ensure the reliability of obstacle avoidance. However, in a complex and narrow environment, such as the veins of the eyeball or the brain or the complex vein environment of plant leaves, Informed-RRT* will fall into a local optimal state, which is not reliable for path searching. Therefore, a fast, effective, asymptotically optimal stochastic path planning algorithm should be introduced. As a high-configuration algorithm, the simple and effective RRT-Connect algorithm requires no adjustment for parameters, which has interactive performance for most situations and improves the speed of tree generation for RRT (Kuffner and LaValle, 2000). Moreover, improvement for the collision-free optimization is further studied. Klemm et al. (2015) combined the ability of RRT-Connect with the best solution of RRT* in order to quickly find a path even in complex environments, which is called the RRT*-Connect algorithm. This method was proven to be successfully faster than normal RRT* and also provided the best solution for convergence.

In addition to route navigation, accurate tracking of trajectory is also an important aspect for microrobot manipulation research (Huan et al., 2022). In order to conduct the application of microrobot to *in vivo* targeted therapy, the external contactless electromagnetically driven system requires a precise controller combined with appropriate control algorithm. Meanwhile, it should be ensured that the actual trajectory of microrobot is appropriate and less drift appeared during the process of movement in the microfluidic environment. Khalil et al. (2014) provided a stable magnetic field and disturbance observation with a double-loop controller, which realized the magnetic flow map construction and point-to-point orientation for the paramagnetic particles. Yang et al. (2018) combined the dynamic model of the microrobot with external perturbations. The motion state of the microrobot was estimated with an extended state observer. Generalized perturbations were compensated as well. Then, a linear trajectory tracking controller was designed based on the estimated motion state to realize the precise manipulation for the microrobot.

In this paper, RRT*-Connect algorithm is introduced for the navigation of a microrobot in a plant vein mimic environment. Based on the planned path, a fuzzy PID controller for trajectory tracking is proposed for precise manipulation in an actual microfluidic environment. Simulation for real-time path planning and trajectory tracking control were conducted in combination with the electromagnetically driven system. The results show that the proposed algorithm and controller can achieve accurate navigation and control for the microrobot manipulation in a branching microenvironment mimicking the plant vein.

## 2 Methods

### 2.1 Force analysis of magnetic drive microrobot

**Figure 1:**
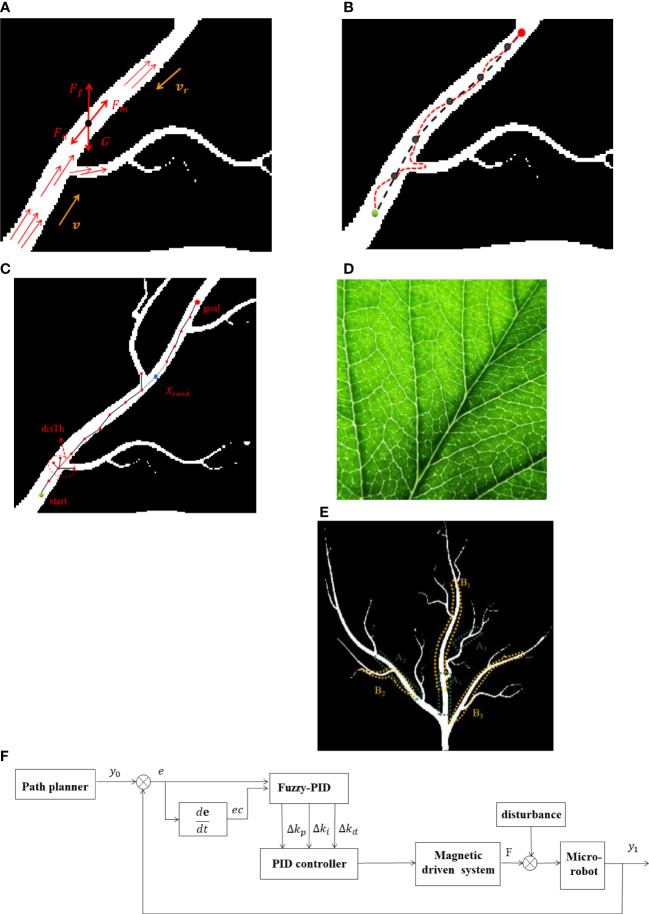
Force analysis and motion trajectory of a microrobot in a microfluidic environment. **(A)** Force analysis of a microrobot. **(B)** Motion trajectory. **(C)** Diagram of RRT*-Connect algorithm. **(D)** Photo of a real plant vein. **(E)** Simulation environment distribution in two conditions. **(F)** Fuzzy PID control diagram.


In our experiment, Fe_3_O_4_ magnetic beads coated with soluble gel were used as microrobots with a diameter of 20 μm. The kinetic analysis for the magnetic microrobot in a microfluidic environment is shown in **Figure 1A**. As soon as the magnetic coils are energized, a gradient magnetic field could be generated in the workspace (Liu et al., 2017; Fan et al., 2022). The induced magnetic force on the microrobot could be given as (1):

(1) $$F_{m}=V(M\cdot \nabla B)=\frac{4\pi R^{3}}{3}(M\cdot \nabla B)$$

(2) $$F_{\text{d}}=-6\pi \eta R\left(v-v_{r}\right)$$

(3) $$G=mg=F_{\text{f}}$$

where *F_m_* is the magnetic force generated by the gradient magnetic field, *M* is the magnetic field strength, ∇*B* is the magnetic field gradient, and *R* is the radius of the microrobot. *F_d_* is the viscous resistance of microfluid, *η* is the fluid viscosity coefficient, *v* is the motion velocity of the robot, and *v*_r_ is the velocity of the microfluid. *G* is the robot’s own gravity, and *F_f_* is the buoyancy of the microrobot.

In order to facilitate the control of the magnetic beads in the fluid environment, the density of the solution is adjusted to be the same as the microrobot. Thus, the gravity and the buoyancy of the microrobot could be ignored. Therefore, the dynamic model of the microrobot in the microfluidic environment is simplified as follows:


(4) $$\int^{}m\frac{d\vec{v}}{dt}=F_{\text{m}}+F_{\text{d}}+\Delta =\frac{4\pi R^{3}}{3}\left(M\cdot \nabla B\right)-6\pi \eta R\left(v-v_{r}\right)+\Delta$$

where *Δ* is the random perturbation in the microfluidic environment.

### 2.2 Navigation and control system design

The flow resistance in the microfluidic environment mainly depends on the radius of the channel and the viscosity coefficient of the fluid, which varies according to the distance to the wall of the micro-channel (Liang et al., 2011). As we can see, at the branch of the plant vein mimic map, the direction of the microfluid flow changes due to the reduction of the leaf vein caliber; therefore, the viscous resistance on the microrobot will also change accordingly. Perturbations could also be generated to enforce the microrobot deviating from the desired trajectory, as shown in **Figure 1B**.

#### 2.2.1 Navigation of microrobot

It has been studied that RRT* algorithm is able to find an initial path quickly. It could be optimized continuously with increasing sampling point within the maximum number of cycles. However, the RRT* algorithm is asymptotically optimized. If a satisfactory path is expected, the time of RRT* algorithm needs to be further optimized. Informed-RRT* algorithm is a sampling optimization process based on the RRT* algorithm, utilizing elliptical sampling instead of global uniform sampling to significantly improve the path search speed. Nevertheless, Informed-RRT* algorithm is prone to fall into local optimum in a relatively narrow environment channel and fail to find a satisfied route. In contrast, RRT*-Connect is able to simultaneously grow two fast random trees for state space search which could improve the path search efficiency. As shown in **Figure 1C**, the start point in the figure is displayed in green, while the end point is displayed in red. During navigation simulation, RRT*-Connect algorithm generates two trees from both start point and end point. It connects the nearest nodes randomly picked from the environment map. For different complexities of various environments, the step size parameter of RRT*-Connect algorithm could be adjusted according to the obtained simulation results. If the time spent for the search process is too long, the step size needs to be increased. On the contrary, if the results are available immediately, the step size could be decreased to get a more optimized path. The maximum step for the search process is denoted by disTh in the RRT*-Connect algorithm. Moreover, the value of disTh could be set flexibly so that all the nodes whose distance is less than disTh are regarded as the same node.

**Figure 1D** shows a real photo of the plant vein which contains lots of branching structures. The simulated map was constructed accordingly. Since the characteristic for different parts of the plant vein mimic map varied, different areas of the map were chosen for the path planning simulation. One series is chosen to be short distance between the start point and end point in different locations (namely, A1, A2, and A3, respectively, for a short distance with a relatively wide, narrow, and narrowest channel) of the vein mimic channel. Another situation is chosen to be a larger distance between the start point and the end point with a different channel width for planning simulation (namely B1, B2, and B3, respectively, for a long distance with a relatively wide, narrow, and narrowest channel), as shown in **Figure 1E**. The width of the channel in the plant vein mimic map gets smaller from the beginning to the end. Afterwards, we compared the searching efficiency with four different algorithms—RRT*-Connect, RRT*, PRM, and Informed-RRT*—in different situations and analyzed the time required for the search process.

#### 2.2.2 Design of fuzzy PID controller

PID is one of the most common algorithms in the field of robot control. The dynamic process could be set faster, smoother, and to be more accurate through the regulation of proportional (P), integral (I), and differential (D). The traditional PID controller is well adapted and has strong robustness. It is a simple algorithm whose control parameter is relatively independent from each other. However, during the trajectory tracking for microrobot manipulation, undesired overshoot could be generated due to the perturbations caused by a variety of microfluidic velocity. In addition, the parameters of the PID controller cannot be adjusted once they are determined. Fuzzy PID controller has been studied widely in the past few decades. It combines the characteristics of a flexible and adaptable fuzzy controller and a highly accurate PID controller. Therefore, this paper adopts a fuzzy PID controller for the manipulation of a microrobot in the plant vein mimic environment, which could make up for the lack of integration link in simple fuzzy controller and solve the problem of fixed parameters in the traditional PID control method.

**Figure 2:**
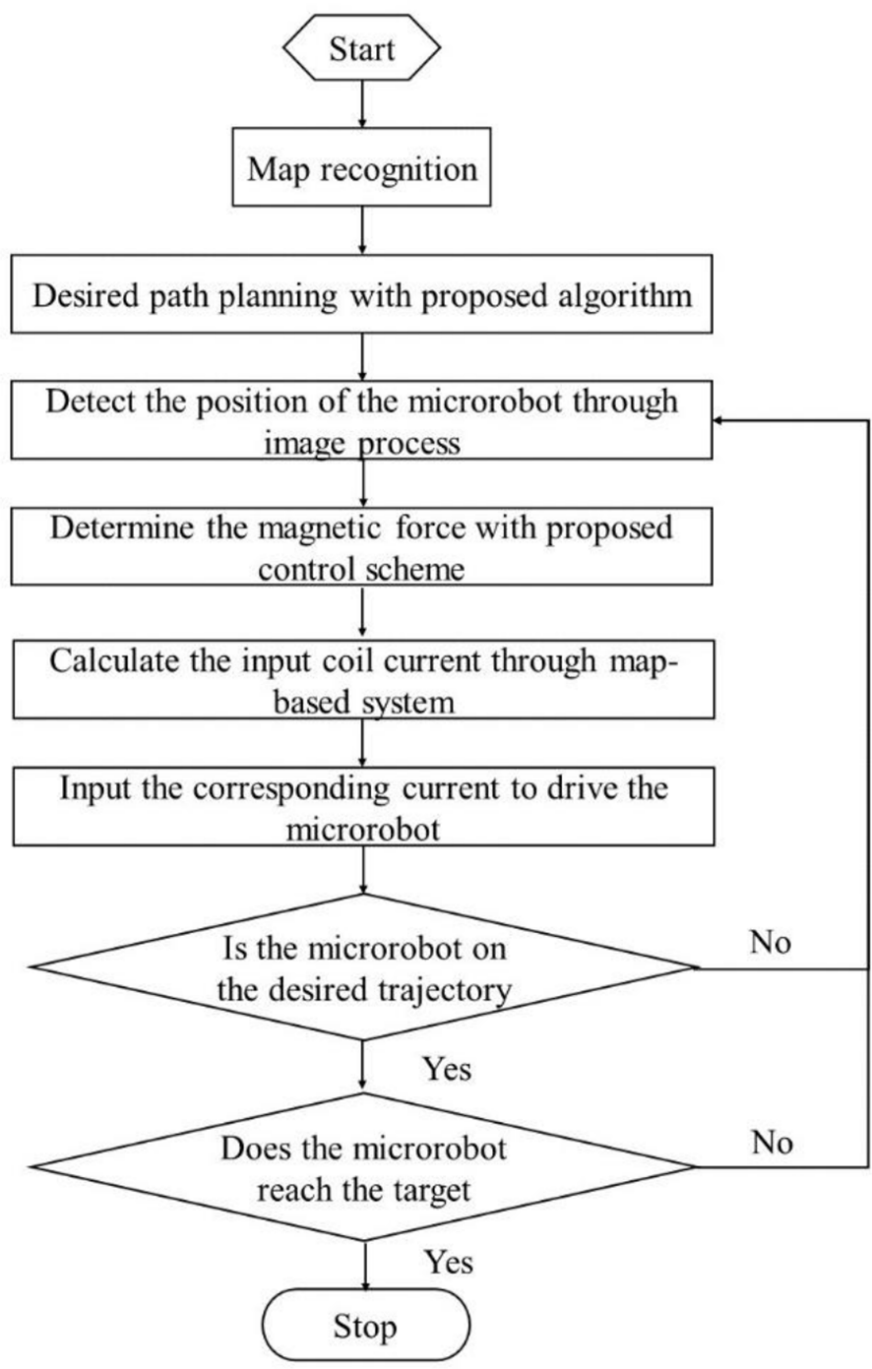
Code architecture of the entire integrated navigation and control system.

According to the trajectory tracking model of microrobot manipulation, the proposed fuzzy controller has two inputs and three outputs. One of the inputs is the displacement error *e* between the actual output movement and the desired trajectory of the microrobot. The other input is the rate of displacement error, given as *ec*. The parameters for a PID controller could be adjusted promptly with a fuzzy controller, which makes the trajectory tracking of a microrobot more accurate. The diagram of the fuzzy PID controller is shown in **Figure 1F**.


(5) $$\text{e}={\text{y}}_{1}-{\text{y}}_{0}$$

(6) $$\text{ec}=\frac{\text{de}}{\text{dt}}$$

(7) $$\{\begin{array}{c} k_{p}=k_{p0}+\Delta k_{p}\\ k_{i}=k_{i0}+\Delta k_{i}\\ k_{d}=k_{d0}+\Delta k_{d} \end{array}$$

(8) $$\text{F}\left(\text{t}\right)={\text{k}}_{\text{p}}\text{e}+{\text{k}}_{\text{i}}\int_{0}^{\text{t}}\text{edt}+{\text{k}}_{\text{d}}\frac{\text{de}}{\text{dt}}$$

where *k_p_*_0_, *ki*_0_, and *kd*_0_ are the initial values of the PID controller, *y*_0_ is the desired trajectory of the microrobot in the microenvironment, *y*_1_ is the actual output trajectory, *e* is the offset error of the robot trajectory, and *ec* is the variation rate of the displacement error. Δ*k_p_*,Δ*k_i_*, and and Δ*k_d_* are the adjustment parameters of the PID controller as well as the output variables of the fuzzy controller. **Figure 2** illustrates the code architecture of the entire integrated navigation and control system. The desired trajectory could be generated with an optimized path planning algorithm. Then, the microrobot was manipulated under the proposed control strategy tracking the preplanned trajectory. During the manipulation, the real position of the microrobot could be detected through image processing. The current input of the experimental setup should be determined by the control system. Thus, a microrobot could be automatically driven to track the preplanned trajectory to the target position.

## 3 Results and discussion

### 3.1 Navigation simulation

In this paper, a plant vein mimic map with a size of 662*612 pixels was constructed. The positions of the start points and the end points for two series of situations mentioned above were pre-set respectively, A1 [start point (345,491), end point (341,406)], A2 [start point (281, 448), end point (217, 381)], A3 [start point (384, 362), end point (419, 332)], B1 [start point (349, 451), end point (371, 224)], B2 [start point (299, 464), end point (183, 399)], and B3 [start point (351, 523), end point (480, 385)]. Each of the two series has a different width of channel for microrobot movement. The basic RRT* algorithm is random, whose search result could be different for the same situation. The average search time used for different algorithms was compared with each other.

**Figure 3:**
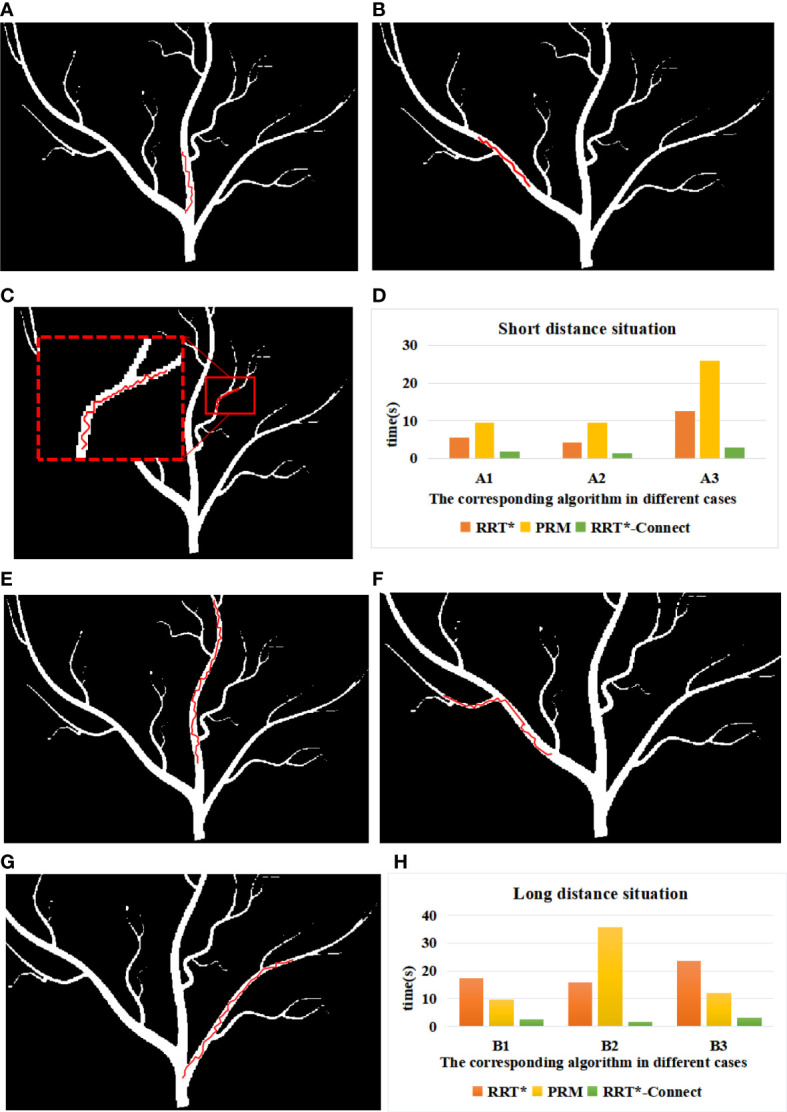
Simulation of road strength planning in case 1: **(A)** A1 environment, **(B)** A2 environment, **(C)** A3 environment, **(D)** B1 environment, **(E)** B2 environment, and **(F)** B3 environment. **(G)** Time consumed by path search in case 1. **(H)** Time consumed by path search in case 2.

As shown in **Figure 3**, the simulation results for the path planning process in the environment described in **Figure 1D** are proposed. The width of the channels gradually decreased as shown in both **Figures 3A–C** and **Figures 3E–G**. According to the simulation results, three kinds of algorithms could smoothly finish the path searching process, either in the relatively wide or narrow channels. As shown in **Figures 3D, H**, data on the time consumption of RRT*, PRM, and RRT*-Connect were collected and compared. It can be seen from the chart that the search speed of RRT*-Connect is relatively faster with a higher success rate compared with other algorithms. Meanwhile, the time consumption of RRT*-Connect is relatively stable in several simulation experiments, which is always the least among the proposed algorithms. This indicates that the RRT*-Connect algorithm could be able to plan a path in a complex environment, with stable probability and fast processing speed. The adaptation ability of the RRT* algorithm was proven to be strong in navigation research, but failure would also appear in some processes of the simulation experiments. PRM is based on the global sampling probability whose time consumption is relatively larger than the other two. Furthermore, when used in situation A3 with the narrowest passage as well as in situation B2 with a relatively narrower channel and a larger traveling distance, the time consumption and failure rate would increase. Informed-RRT* algorithm has also been utilized for navigation in the plant vein mimic map, which was used in our previous research (Huan et al., 2022). This algorithm shrinks the sampling space into an oval area based on a feasible path obtained by RRT*. However, as the microrobot moved into the narrow channel of the map, the Informed-RRT* algorithm would fall into a locally optimal solution. Thus, in most of the times in our simulation experiments, Informed-RRT* failed to find a suitable route in a limited time. By comparing the results of the simulation of the RRT*, PRM, and RRT*-Connect algorithms, it can be concluded that the RRT*-Connect algorithm is highly adaptable and the most stable among the proposed algorithms.

### 3.2 Trajectory tracking analysis

**Figure 4:**
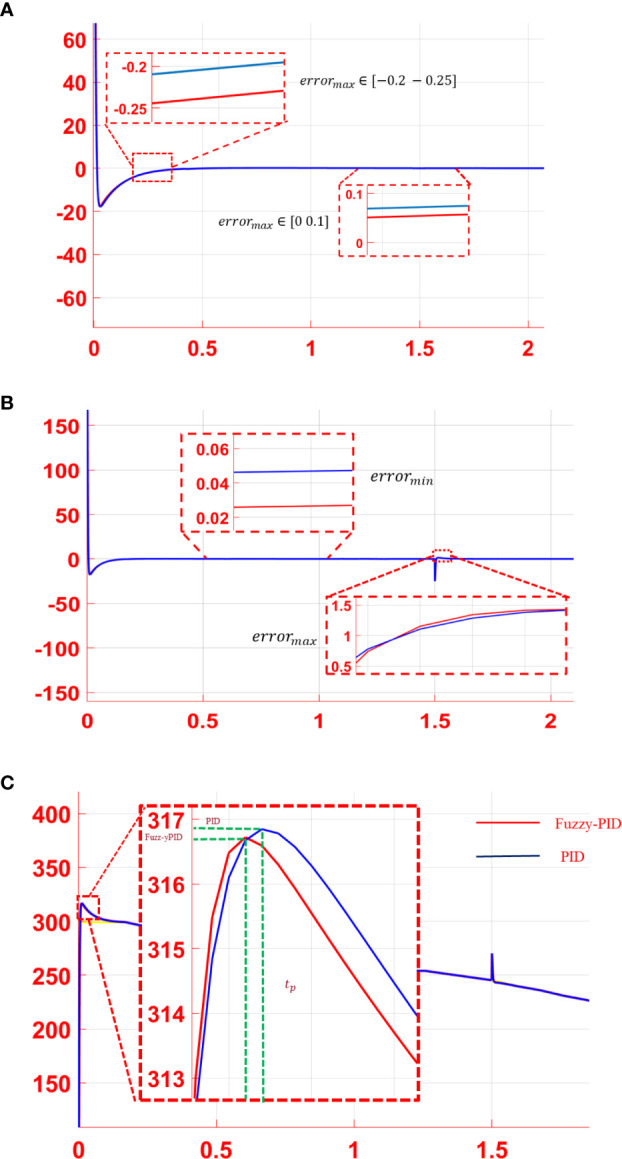
Trajectory tracking error of the PID controller (blue curve) and the fuzzy PID controller (red curve). **(A)** Tracking error without disturbance. **(B)** Tracking error with step signal disturbance. **(C)** Control performance analysis with step disturbance.

In practice, the movement of a microrobot would be affected by the disturbance of the microfluidic environment factor, which leads the actual trajectory to deviate from the desired trajectory. Therefore, a suitable controller should be introduced to adjust the actual error of the trajectory as close to the preplanned trajectory as possible in the plant vein mimic map. To make the process more reliable, the environment B2 with vein branches and a long distance between the start and end points was tested. Tracking simulation experiments were conducted both with and without step disturbances. Moreover, the trajectory tracking error was analyzed, as shown in **Figure 4**. The red line in the figure indicates the control curve of the fuzzy PID controller, while the blue line stands for the traditional PID controller with proper parameters. As illustrated in **Figures 4A, B**, the PID controller and the fuzzy PID controller have a similar capability to keep the desired trajectory for microrobot manipulation with a small tracking error. According to the tracking curve, the response speed of the PID controller and the fuzzy PID controller at a certain time could be different after a step signal disturbance was introduced. As proposed in **Figure 4C**, the control performance with step disturbance was analyzed. The peak value and overshoot of the fuzzy PID controller are a bit less than those of the traditional PID controller. Thus, the control effect of the fuzzy PID is relatively better than the PID controller.

In the microfluidic environment, the movement microrobot is mainly influenced by the flow velocity. The viscous resistance increased as the microrobot moved closer to the channel wall. That is to say, flow velocity varied in different positions of the flow channel. Thus, step signal disturbance cannot simulate the real environment exactly. A sinusoidal periodic signal, as given in Eq. 9, was added to the system as the disturbance for further processing:


(9) $$y=10\text{sin}\left(30\text{x}+\frac{\pi }{3}\right)$$

**Figure 5:**
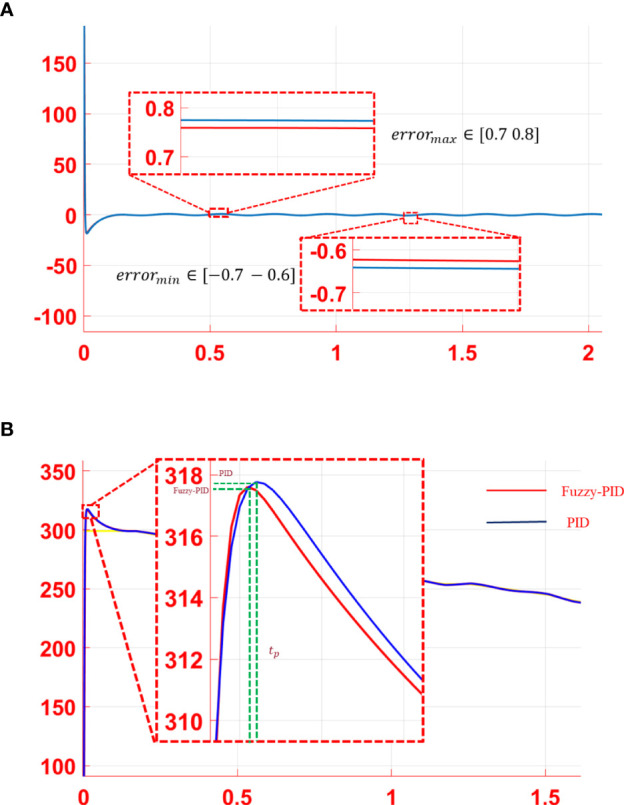
Analysis of the simulation results: **(A)** error curve and **(B)** performance analysis of tracking curve.

With the proposed sinusoidal periodic disturbance, the tracking error curves and the tracking performance of the PID controller and the fuzzy PID controller were performed, as shown in **Figure 5**. According to the tracking error curves in **Figure 5A**, both the PID controller and the fuzzy PID controller have a good anti-interference ability in the trajectory tracking process. However, the steady-state error of the fuzzy PID controller is less than that of the PID controller. As shown in **Figure 5B**, compared with the PID controller, the system overshoot of the fuzzy PID controller is also smaller than that of the PID controller, which indicates a better controller performance. The mean squared tracking errors for two series of situations are illustrated in **Table 1** under the two different control methods. In conclusion, the proposed fuzzy PID controller can realize the secondary regulation for the system on the basis of improving the accuracy of the traditional PID controller. The disturbance caused by the microfluidics could be adjusted effectively.

**Table 1:** Mean square of the tracking errors.

Simulation number	Control scheme	Position error (pixel)
A1	PID	0.59
A2		0.68
A3		0.83
B1		0.65
B2		0.71
B3		0.68
A1	Fuzzy PID	0.43
A2		0.54
A3		0.55
B1		0.58
B2		0.63
B3		0.49

## 4 Conclusion

In this paper, an integrated navigation and control system for a microrobot was proposed. A dynamic model of a microrobot was analyzed within the microfluidic environment. The desired trajectory of the microrobot was planned through the RRT*-Connect algorithm, which has a better performance than other similar algorithms. In order to further improve the driven accuracy for manipulating a microrobot to move along the desired trajectory, a fuzzy PID controller was introduced. Based on the online parameter adjustment of the PID controller with a fuzzy module, the trajectory tracking error for a microrobot could be reduced, and the actual movement is much closer to the desired trajectory. The simulation results show that the proposed integrated system can significantly improve the anti-interference ability and track the tracking accuracy of microrobots in a microfluidic environment mimicking the plant vein. It lays the foundation for further promoting the plant/organism application of microrobots. The microrobots manipulated in the plant vein could be used as microsensors to study the dynamics of plants under photosynthesis. In addition, the bio-microsensors could monitor the health of the plant in real time.

## Data availability statement

The original contributions presented in the study are included in the article/supplementary material. Further inquiries can be directed to the corresponding author.

## Author contributions

ZH, JW, and WM: conceptualization, methodology, validation, data analysis, and writing—original draft preparation. LZ, ZZ, and ZC: material development and writing—review and editing. All authors contributed to the article and approved the submitted version.
